# Venom gland transcriptomics and bioactivity profiling suggest bifunctional hyaluronidase activity in the venom of *Mesobuthus crucittii* (Scorpiones: Buthidae)

**DOI:** 10.3389/fmolb.2026.1807239

**Published:** 2026-06-01

**Authors:** Masoumeh Baradaran, Fatemeh Salabi, Nasrin Payab, Seyed Mahdi Kazemi, Carlos E. Santibáñez-López, Tim Lüddecke

**Affiliations:** 1 Toxicology Research Center, Medical Basic Sciences Research Institute, Ahvaz Jundishapur University of Medical Sciences, Ahvaz, Iran; 2 Razi Vaccine and Serum Research Institute, Agricultural Research, Education and Extension Organization (AREEO), Ahvaz, Iran; 3 Graduate of the Department of Biology, Faculty of Science, Shahrekord University, Shahrekord, Iran; 4 Zagros Herpetological Institute, Qom, Iran; 5 Department of Biology, Western Connecticut State University, Danbury, CT, United States; 6 Fraunhofer Institute for Molecular Biology and Applied Ecology, Gießen, Germany

**Keywords:** drug discovery, hyaluronidase, *Mesobuthus crucittii*, scorpion venom gland, transcriptomic analysis

## Abstract

**Introduction:**

Scorpion venom is a rich source of bioactive molecules with promising biomedical applications. Hyaluronidases are venom-associated enzymes that facilitate toxin diffusion by degrading extracellular matrix glycosaminoglycans, yet their structural diversity and substrate specificity in scorpion venoms remain insufficiently explored. This study aimed to identify and characterize a novel hyaluronidase from the venom gland of the Iranian endemic scorpion *Mesobuthus crucittii* and evaluate its biochemical and structural properties.

**Methods:**

Venom gland transcriptome profiling was performed. Hyaluronidase sequences were analyzed *in silico* via phylogenetic reconstruction, motif prediction, physicochemical property calculation, and structural modeling. Molecular docking was conducted to explore interactions with hyaluronic acid and chondroitin sulfate substrates. Enzymatic activity and thermal stability were experimentally evaluated using turbidimetric assays.

**Results:**

Transcriptomic analysis revealed a diverse toxin repertoire dominated by ion channel modulators and enzymatic components, including a novel precursor encoding a putative hyaluronidase exhibiting a unique cysteine framework with six disulfide bonds and three conserved diagnostic motifs (GDWW, FPDC, and GWGS). Structural modeling suggested catalytic and binding domains consistent with glycosyl hydrolase family 56 enzymes. Molecular docking supported preferential binding affinity toward hyaluronic acid tetrasaccharides rather than longer or highly sulfated glycosaminoglycans. Functional assays on crude venom confirmed strong hyaluronan degradation and slower chondroitin sulfate hydrolysis, indicating dual substrate activity.

**Discussion:**

This study supports the existence of a structurally distinct scorpion venom hyaluronidase with putative bifunctional substrate activity. These findings expand current understanding of scorpion venom enzyme evolution and highlight the enzyme's translational potential.

## 1 Introduction

Scorpions are a lineage of arthropods comprising nearly 2,900 described species distributed worldwide across 24 families (Santibáñez-López et al., 2023; Rein, 2023). The Middle East is one of the regions with the highest scorpion diversity globally (Lourenço, 2015), and Iran is recognized as a hotspot for scorpion diversity (Ward et al., 2018; Kazemi and Sabatier, 2019; Salabi, 2025a; Kazemi et al., 2024). Currently, 99 scorpion species classified into 20 genera and four distinct families have been identified in Iran (Kazemi and Sabatier, 2019; Salabi et al., 2025b; Kazemi et al., 2024; Baradaran et al., 2024). Several scorpion species cause envenomations that may lead to fatalities, particularly in southern and southwestern regions of the country (Safdarian et al., 2022; Ward et al., 2018). Annually, the Iranian Ministry of Health reports various symptoms associated with scorpion stings, encompassing local pain, sweating, vomiting, delirium, anxiety, necrosis, inflammation, muscle paralysis, hematuria, and prolonged edema. Numerous studies have been undertaken to elucidate the primary contributing factor behind these symptoms, with a predominant focus on identifying venom compounds (Ghorbani et al., 2021; Salabi, 2025a). Understanding scorpion venom is crucial for two primary reasons: first, to elucidate the mechanisms underlying its pathological effects in envenomated individuals, and second, to explore its potential for therapeutic applications. Detailed characterization of venom proteins not only reveals the functional roles of toxins but also provides a vital foundation for the development of novel pharmaceuticals.

The venom of scorpions has been extensively studied to identify transcripts encoding specific toxic peptides responsible for the neurotoxic effects observed during envenomation or even non-toxic peptides. These venom peptides are important for both medical and pharmaceutical applications (Baradaran et al., 2024; Possani et al., 1999; Salabi and Jafari, 2023). Scorpion venom consists mainly of a mixture of proteins and peptides with various biological functions. Some of its toxic compounds, which play a vital role in the survival of scorpions, are used for hunting prey, defense against predators, and reproductive purposes. Others including metalloproteases, serine proteases, phospholipases A_2_, lipolysis activating peptides (LVPs), hyaluronidase, antimicrobial peptides, and ion channels peptides, are considered promising candidates in the venoms-to-drugs pipeline (Pessini et al., 2001; Morey et al., 2006; Abdel-Rahman et al., 2013). To date, various powerful strategies have been employed for rapid analysis of numerous venom components. Among these, transcriptomic analysis is known to be an important tool for studying the global composition of venom through mRNA expression profiles and their relative expression levels (Vonk et al., 2021). A key strength of transcriptomic analysis, compared to proteomic methods, is its capacity to provide insights into patterns of gene activation (*via* read counts) and gene evolution (*via* coding sequences) (Vonk et al., 2021). To mitigate the inherent overestimation of toxin diversity by transcriptomics, contemporary studies increasingly favor a combined transcriptomic and proteomic (“multi-omics”) strategy for a more complete understanding. Hyaluronidase, an enzyme common across arachnid venoms, has recently gained attention (Dresler et al., 2025; Dresler et al., 2024a; Dresler et al., 2024b). It has emerged as a promising medical adjuvant that enhances drug absorption into tissue, reduces tissue damage, and correct complications and unsatisfactory results after hyaluronic acid filler injections. Three forms of commercially available hyaluronidase are testicular hyaluronidase extracted from bovine or ovine sources, and human recombinant hyaluronidase (Borzabadi-Farahani et al., 2024).

Considering that hyaluronidase is commonly found in animal venoms, these venoms represent potential novel sources for enzyme production. Venom hyaluronidase has been identified as an allergen capable of inducing severe and fatal IgE-mediated anaphylactic reactions in humans (Bordon et al., 2015). Hyaluronidase from arthropod venom plays a critical role in systemic envenomation, particularly immune system stimulation and inflammatory responses. These enzymes hydrolyze hyaluronic acid and chondroitin sulfates into smaller oligosaccharides, acting as pro-inflammatory mediators (Moreno and Giralt, 2015). The interaction of venom toxins with hyaluronic acid in the extracellular matrix increases tissue permeability during envenomation, facilitating toxin diffusion (Salabi and Jafari, 2023). Hyaluronidases extracted from several vespinae species and spider venoms have been shown to depolymerize hyaluronic acid and, to a lesser extent, chondroitin sulfate (Allalouf et al., 1972; Bordon et al., 2015). While the role of scorpion venom hyaluronidases in envenomation is well documented (Horta et al., 2014; Oliveira-Mendes et al., 2019), their comprehensive biochemical characterization and activity against chondroitin sulfates remain insufficiently explored. To address this knowledge gap, we analyzed the venom gland transcriptome of the Iranian endemic scorpion *M. crucittii* using RNA sequencing, focusing on the *in silico* characterization of venom hyaluronidases. Additionally, we investigated the enzymatic activity of *Mesobuthus crucittii* venom hyaluronidase against both hyaluronic acid and chondroitin sulfate substrates using a turbidimetric assay. These findings indicate that this enzyme exhibits dual hydrolytic activity toward both substrates, reinforcing previous observations of scorpion hyaluronidase activity against chondroitin sulfate.

## 2 Materials and methods

### 2.1 Sample preparation and RNA sequencing

Scorpions were manually collected from the deserts of the Khuzestan province in southwest Iran, and subsequently brought to the Razi Vaccine and Serum Research Institute laboratory. Three days before RNA extraction procedure, the scorpion venom was extracted by electrostimulation to stimulate the expression of venom components. Ten specimens were euthanized to dissect their telsons 3 days following the venom extraction. These telsons were powdered using liquid nitrogen. All procedures performed in this study were under the ethical principles and the national norms and standards for conducting Medical Research in Iran and were authorized by the Institutional Animal Care Committee of Razi Vaccine and Serum Research Institute (Permit number IR. RVSRI.REC.1401.017) and Ahvaz Jundishapur University of Medical Sciences (Ethical code: IR. AJUMS.REC.1400.557). Total RNA was extracted using the RNeasy Animal Mini Kit (Qiagen, Valencia, CA, USA) following the manufacturer’s protocol. Two mRNA libraries comprising three RNA extractions pooled were then sequenced *via* Illumina HiSeq 2000 platform at Macrogen Co. (Seoul, Korea).

### 2.2 Transcriptome assembly and annotation

For transcriptome analysis, we implemented previously established workflows for venom gland transcriptomes (e.g., Lüddecke et al., 2025; Hurka et al., 2022). Quality of raw reads was determined using FastQC (Wingett and Andrews, 2018), with poor quality reads trimmed using Trimmomatic (Bolger et al., 2014) following our previous protocols (Salabi, 2025a; Baradaran and Salabi, 2023). Selected reads were then assembled into transcripts using the Trinity software v2.15.1 with the following parameters: -normalize_reads, --seqType fa, --SS_lib_type RF, --max_memory 32G, --CPU 8. Qualified reads from the two pooled samples were subjected to *de novo* assembly, resulting in the generation of a unique transcriptome (Raw assembly). To reduce sequence redundancy from the Trinity assembly, we used CD-HIT-EST v4.7 (Li and Godzik, 2006) resulting in a new assembly (Final assembly). To assess the quality of both assemblies, we ran the Trinity script ‘TrinityStats.pl’. To evaluate the quality of the clustered *de novo* transcriptome assemblies, we counted the percentage of orthologues conserved across the Arachnida_odb10 dataset using BUSCO (Manni et al., 2021). Lastly, to detect cross-contamination in our transcriptomes, we used CroCo v0.1 software (Simion et al., 2018). To annotate our assemblies, we used the TransDecoder pipeline (Haas et al., 2013), with the “single best ORF” option selected to predict open reading frames (ORFs) and retrieve untranslated regions and coding sequences. Next, the Trinotate v4.0.2 pipeline facilitated transcriptome assembly annotation. Full-length venom protein sequences were identified using BLASTX or BLASTP by alignment against manually reviewed toxin databases from the UniProtKB Animal toxin annotation project. Lastly, we implemented the tapai pipeline (Santibáñez-López et al., 2022) to validate the annotation of scorpion toxins. The raw data were deposited into National Center for Biotechnology Information (NCBI) Sequence Read Archive (SRA) database with the BioProject ID PRJNA1233588.

### 2.3 Hyal *in silico* characterization

Hyal transcripts retrieved from the annotation pipeline were compared against additional scorpion Hyal sequences retrieved from UniProt or published databases (Salabi et al., 2021) (Supplementary Table S1). These sequences were aligned using MAFFT v.7.407 (Katoh and Standley, 2013) with default parameters. To test relationships of these Hyal homologs, a gene tree was constructed implementing the Maximum Likelihood approach using IQ-TREE v. 2.0.6 (Minh et al., 2020) with ModelFinder Plus (Kalyaanamoorthy et al., 2017) for automated model fitting and nodal support values assessed with ultrafast bootstrap replicates (Hoang et al., 2018). To characterize the signature of bioactivity in Hyal sequences, the conserved amino acid motifs, the number of cysteines, and disulfide bonds of the obtained sequences corresponding to Hyal were predicted using the InterProScan search program, the Simple Modular Architecture Research Tool (SMART), PROSITE, and Disulfide by Design 2.0 web-based tool (Craig and Dombkowski, 2013). Signal peptides within the amino acid sequences were identified using the SignalP6 server. The physical and chemical characteristics, including molecular weight, amino acid count, theoretical isoelectric point (pI), number of positively charged (Arg + Lys) and negatively charged (Asp + Glu) amino acid residues, amino acid composition profile (%), instability index, estimated half-life, and grand average of hydropathicity (GRAVY) value of the Hyal proteins, were determined using the ProtParam server provided by Expasy. Mapping specific antibody–antigen interactions of Hyal of *M. crucittii* was measured using the Antibody Epitope Mapping server (AbEMap) and Bepipred Linear Epitope Prediction 2.0.

### 2.4 Molecular docking

The amino acid sequence of Hyal from *M. crucittii* was used as a query to perform a protein BLAST search against the UniProt database (https://www.uniprot.org/blast). This search identified Hyal one from *Olivierus martensii* (UniProt accession: P86100) as the closest homolog. The putative ligand-binding site was identified based on UniProt data for protein P86100 and sequence analysis of the enzyme. Subsequently, post-translational modifications (PTMs) for the *M. crucittii* Hyal sequence were predicted using the PTMGPT2 server (https://nsclbio.jbnu.ac.kr/tools/ptmgpt2) (Shrestha et al., 2024).

The three-dimensional structure of *M. crucittii* Hyal was modeled using the AlphaFold server (https://alphafoldserver.com) (Jumper et al., 2021) as described previously (Dersch et al. 2026). Briefly, the quality of the generated models was rigorously evaluated using the SAVES server (https://saves.mbi.ucla.edu/), which integrates multiple validation tools including ERRAT, Verify3D, and PROCHECK (Laskowski et al., 1993; Lüthy et al., 1992; Colovos and Yeates, 1993). Furthermore, stereochemical quality was assessed *via* Ramachandran plot analysis using MolProbity (http://molprobity.biochem.duke.edu/). The model with the highest overall validation scores was selected for all subsequent analyses, including the structural comparison and molecular docking. The selected tertiary structure of *M. crucittii* Hyal was used to perform a comparative structural alignment against the homologous structure from *O. martensii* and the crystal structure of Hyal from *Apis mellifera* venom (PDB ID: 1FCQ). All structural alignments and visualizations were conducted using UCSF Chimera software v1.11.2 (https://www.cgl.ucsf.edu/chimera/).

Molecular docking was performed with two glycosaminoglycan substrates, hyaluronic acid (HA) and chondroitin sulfate A (CSA), to investigate the substrate-binding affinity of the enzyme. The three-dimensional structures of the ligands were obtained from the Protein Data Bank (PDB). The hexasaccharide structure of HA (PDB ID: 1HYA) was used, and a tetrasaccharide form was generated by removing the two terminal monosaccharide units. Similarly, a tetrasaccharide form of CSA was generated from its crystal structure (PDB ID: 1C4S) using the same method. The selection of tetrasaccharide and hexasaccharide forms for docking was based on the findings of Jiang et al. (2011), which identify these oligomers as the predominant hydrolysis products of Hyal activity. The putative ligand-binding site for docking simulations was defined based on two key factors: (i) annotation in the UniProt database (entry P86100) and (ii) the presence of conserved catalytic motifs identified within the enzyme sequence in the current study. This region is, therefore, predicted to encompass the catalytic core and key substrate-binding residues. Molecular docking of the modeled Hyal with tetrasaccharide and hexasaccharide forms of hyaluronic acid and chondroitin sulfate A was performed using AutoDock 4 (Morris et al., 2009). PDB files of the enzyme and ligands were converted to PDBQT format using AutoDockTools. The binding region was defined as a grid box covering amino acids 120 to 140, and the Lamarckian Genetic Algorithm (LGA) was executed using default parameters. Binding energies, ligand orientation, and interaction types (hydrogen bonds, hydrophobic contacts, electrostatic interactions) were analyzed.

### 2.5 Hyaluronidase assay

The protein content of the *M. crucittii* crude venom was quantified using the Bradford assay (He, 2011; Nazari, 2024), with bovine serum albumin (BSA) as the standard. Absorbance measurements were taken at 595 nm. The Hyal activity of crude venom was monitored using a Single Beam Scanning M501 spectrophotometer (UK). Testicular Hyal and bee venom Hyal were employed as positive controls due to their well-known activity toward hyaluronan and chondroitin sulfate (Nazari et al., 2023). Lyophilized crude venom from Iranian honeybees was purchased from the Agricultural Science and Natural Resources, University of Khuzestan (Mollasani, Iran), and ovine testis Hyal was purchased from Wockhardt (UK). Additionally, hyaluronic acid sodium salt, hexadecyltrime-thylammonium bromide, and a mixture of chondroitin 4-sulfate (60%) and 6-sulfate (40%) were purchased from Sigma Chemical Co. (St. Louis, MO, USA). Bradford Reagent (BioShop, Burlington, ON, Canada, BRA222) was used for analytical purposes. The activity of venom Hyal toward hyaluronan or chondroitin sulfate substrates was assayed using a turbidimetric method (Nazari et al., 2023, Nazari et al., 2024). Briefly, 1 mg of each lyophilized crude venom was dissolved in 1 mL of sodium acetate buffer (pH 6) and shaken for 2 h at room temperature. Ovine Hyal was prepared according to the company’s protocol (1500 I.U. × 1 mL sodium acetate buffer). Then, 50 µL of each enzyme solution (bee, scorpion, ovine) was added to 100 µg of hyaluronic acid or 500 µg of chondroitin sulfate. The final volume was set to 1 mL with 0.2 M sodium acetate buffer (pH 6.0). Reaction mixtures containing hyaluronic acid were incubated at 37 °C for 15–30 min. For chondroitin sulfate, reactions were incubated at 37 °C for 1–120 h. Degradation was monitored by measuring optical density at 400 nm. The data presented represent relative enzymatic activity expressed as a percentage (%).

## 3 Results

### 3.1 De novo transcriptome assembly and annotation

Following adapter and low-quality reads trimming processes, a total of 44370955 paired 150 bp clean reads were successfully retained. Trinity *de novo* assembling yielded 206153 contigs greater than 200 bp in length. Assembly of contigs produced 135021 trinity genes with a median transcript length of 436 (Supplementary Table S1). Mapping the cleaned reads back to their corresponding assembly using Bowtie2 v2.3.4.1, resulted in >98.23% mapped reads. The transcripts from the Trinity assembly were clustered together using CD-Hit-EST with a stringent similarity threshold of 0.95. The quality of transcriptome generated using Trinity (Raw transcriptome) or resulting from the clustering step (Final transcriptome) was evaluated using the Trinity toolkit, BUSCO v 3.0.2, and Croco v0.1 packages. The results indicated the *de novo* assembly of all reads in a total assembly of 63, 121, 168 bp, representing 97,421 transcripts with an N50 size of 1,968 bp corresponding to 93,303 trinity genes (Supplementary Table S2). The CD-Hit-EST clustering reorganized the Trinity genes into 93,303 unigenes, representing a 69.1% reduction in the number of unigenes in the final assembly compared to the raw assembly (Supplementary Table S3). In this respect, CD-HIT performed well in clustering similar biological sequences together.

The number of complete, duplicated, fragmented, and missing transcripts were also calculated in each of our *de novo* assemblies using BUSCOs v5.2.2 against the Arachnida gene set. The raw assembly demonstrated high completeness, identifying 2,711 (92.4%) complete genes out of 2,934 conserved Arachnida genes. This included 252 (8.6%) complete single-copy and 2,459 (83.8%) complete duplicated BUSCOs, along with 86 (2.9%) fragmented and 137 (4.7%) missing genes. Subsequent clustering significantly reduced duplication, resulting in a final assembly with 2,172 (74%) complete single-copy and 494 (16.8%) complete duplicated BUSCOs, indicating improved assembly quality. The final assembly also contained 109 (3.7%) fragmented and 159 (5.5%) missing BUSCOs. Furthermore, Croco v0.1 analysis confirmed the absence of contamination.

**FIGURE 1:**
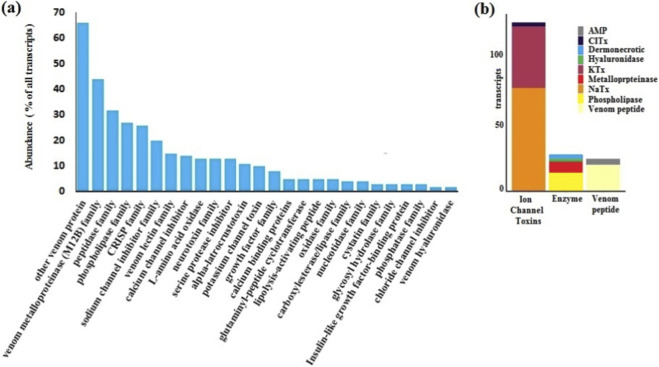
Abundances of precursors in *Mesobuthus crucittii* venom gland transcriptome. **(a)** Relative abundance of venom protein families, expressed as a percentage of total toxin transcripts, identified through annotation against the Animal Toxin Annotation Project database. **(b)** Distribution of *Mesobuthus crucittii* venom gland transcripts validated by *tapai*.

To identify venom components, we annotated the *M. crucittii* venom gland transcriptome against the UniProtKB/Swiss-Prot animal toxin database. This annotation revealed transcripts corresponding to 26 distinct venom component families, notably ion channel modulators, metalloproteinases, peptidases, phospholipases, and cysteine-rich secretory proteins (CRISPs) (Figure 1a). To validate our toxin annotation, we used the neural network approach implemented in *tapai*. We trained *tapai* using the two default models (scorpion venom components and housekeeping genes), and the amino acid sequences annotated with blastp. From 177 selected transcripts (Supplementary Material S1): 126 were classified by tapai as ion channel toxins, 27 as enzymes and 24 as other venom components (e.g., CRISP, and other venom peptides; Figure 1b).

**FIGURE 2:**
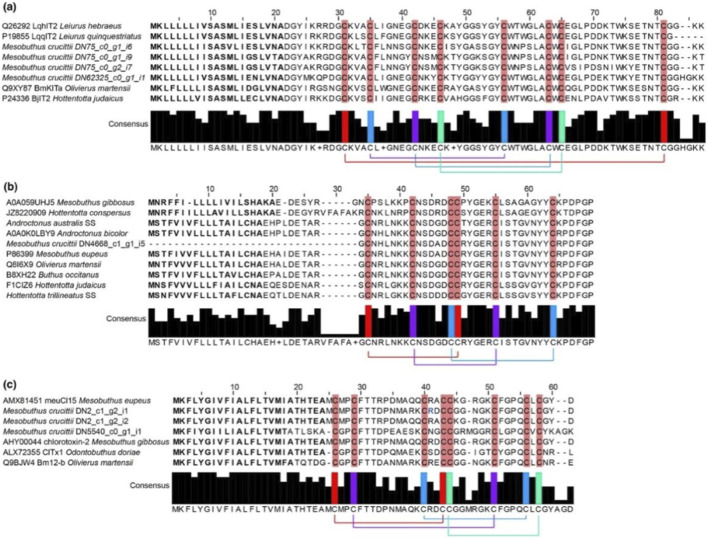
Multiple sequence alignment (MSA) of the validated selected NaTx **(a)**, KTx **(b)**, and ClTx **(c)** from the venom gland transcriptome of *Mesobuthus crucittii*. Consensus sequence histograms of each clade below the MSA with highly conserved disulfide bridges (lines) formed by six or eight cysteines.

Within the ion channel toxins, *tapai* classified 77 transcripts as putative sodium channel toxins (NaTx), 46 potassium channel toxins (KTx) and 3 chloride channel toxins (ClTx). Salient among the ion channel toxins validated by tapai, we found: (a) four transcripts with identities in between 69%–86% to the Beta-insect depressant toxin LqhIT2 (NaTx) from *Leiurus quinquestriatus* (UniProt accession P19855; Figure 2a), one transcript had a 92% of identity to the lambda MeuTx (KTx) from *Mesobuthus eupeus* (P86399; Figure 2b), and three transcripts with 75%–100% identity to the Chloride channel toxin meucCl15 (ClTx) from *M. eupeus* (AMX81451; Figure 2c).

### 3.2 Phylogenetic analysis and *in silico* molecular characterization

**FIGURE 3:**
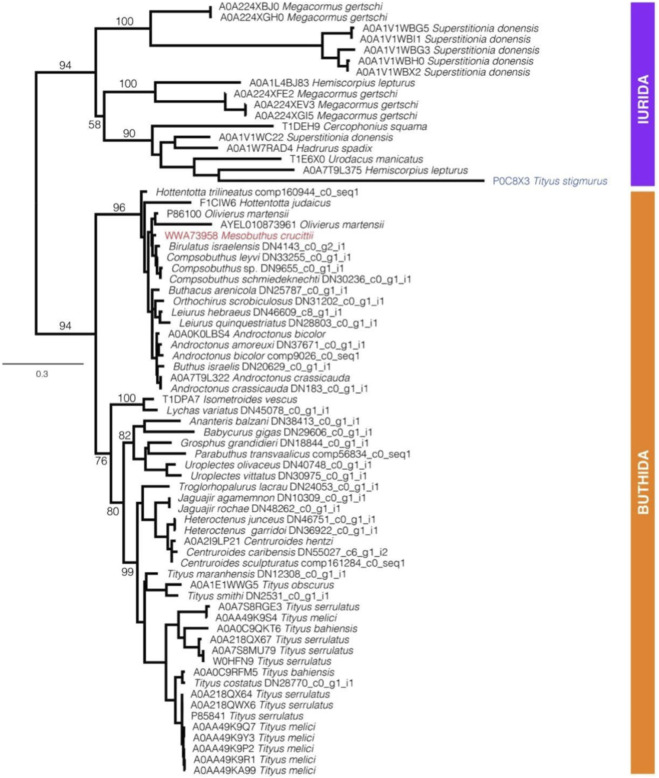
Maximum likelihood gene tree (midpoint rooted) of 71 scorpion hyaluronidase (Hyal) sequences. In red, *Mesobuthus crucittii* Hyal, with the single buthid Hyal sequence (P0C8X3) within the predominantly Iurid clade in blue. Numbers above selected branches indicate nodal support.

Our venom gland transcriptome of *M. crucittii* venom gland, validated by *tapai*, identified two transcripts with 96% identity to the Hyal one from *O. martensii* (P86100). The resulting consensus sequence was deposited in GeneBank and to the ScorpDB (Baradaran et al., 2024) under the Accession numbers PP347720 (mRNA sequence) and WWA73958 (protein sequence). The amino acid sequence of newly identified Hyal of *M. crucittii* was aligned to 71 other Hyal sequences isolated or sequenced from different scorpion species (Supplementary Table S4). Maximum likelihood phylogenetic reconstruction *via* IQ-TREE retrieved a topology of the scorpion Hyal sequences that largely mirrored the scorpion tree of life (Figure 3). Two major monophyletic clades were recovered with strong support (94%): One exclusively containing buthid Hyals, and the other primarily comprising iurid Hyals, with a single buthid sequence as an exception (P0C8X3, *Tityus stigmurus*). Within the buthid clade, five well-supported clades were identified, consistent with internal buthid phylogeny. Notably, *M. crucittii* Hyal grouped within a clade containing only species from the “*Buthus*” group, which was sister to a clade comprising sequences from the “*Isometrus*” “*Uroplectes*” “*Ananteris*” and “*Tityus*” groups.

**FIGURE 4:**
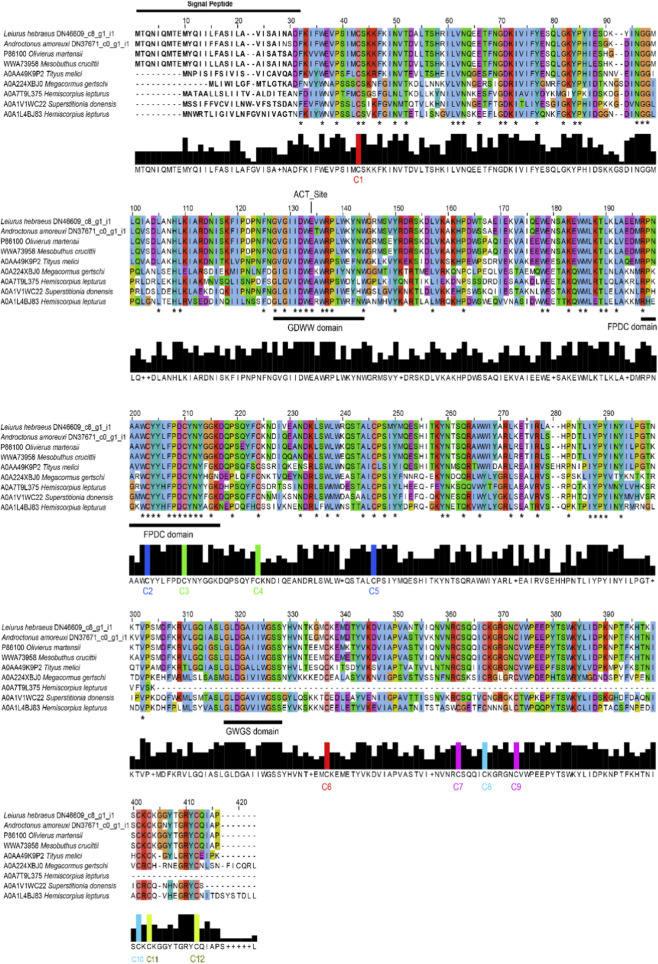
Multiple sequence alignment (MSA) of the selected Hyal sequences from the venom gland transcriptome of *Mesobuthus crucittii* and other related scorpion species (see also Figure 3). Signal peptide is indicated with a black straight line, conserved invariant residues are shown by stars, active site (ACT) of Hyal indicated by a line. Consensus histograms of each clade below the MSA with highly conserved disulfide bridges (lines) formed by 12 cysteines color coded by pairs.

Based on the Multiple Sequence Alignment (MSA), we found that profiles of Hyal family had three highly conserved segments (Figure 4; Supplementary Figure S1). All Hyal sequences from scorpions are characterized by three motifs; Gx^3^IDWExWRPxWx^3^W, RPx^3^WxYYxFPDCYxG, and Gx^2^Gx^3^WGSS, for which three domains termed GDWW, FPDC, and GWGS can be defined respectively. The Hyal of *M. crucittii* contains twelve cysteine residues, which leads to the formation of six disulfide bridges. These six disulfide bonds are stacked in the order C1−C6, C2−C5, C3−C4, C7−C9, C8−C10, and C11−C12 (Figure 4; Supplementary Figure S1). Prior to this study, no significant sequence homology had been reported for the C-terminal domains of scorpion Hyals. Here, we found a region (55–62 amino acid residues) containing a cysteine-rich pattern, CSx^3^Cx^3^Gx^1^Cx2Px^6−7^Wx^9-10^Fx^4^Ix^1^Cx^1^Cx^4−5^Gx^2^C (where the subscripts indicate the spacing between the cysteine residues and the “x” denotes any residue). This pattern is identified in the InterProScan database as an epidermal growth factor EGF-like domain that consist of three disulfide bonds which stacked in the order C7−C9, C8−C10, and C11−C12.

**FIGURE 5:**
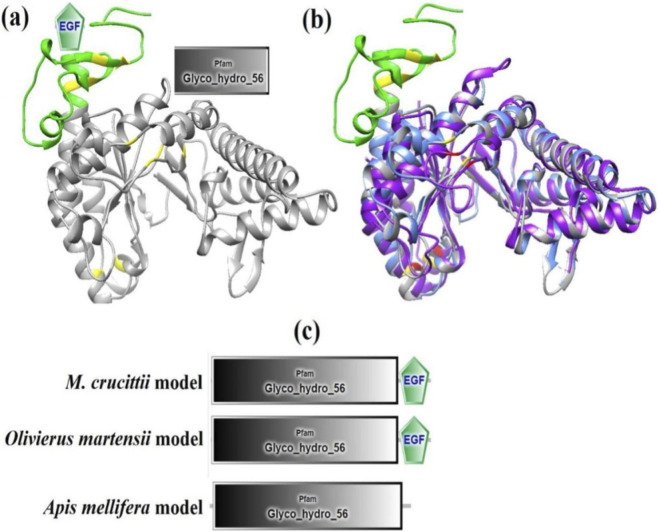
3-D structure of Hyal of *Mesobuthus crucittii* model **(a)**, structural alignment of Hyal from *Mesobuthus crucittii*, DB model of Hyal-1 from *Olivierus martensii* (P86100.1), and crystal structure (Monoclinic) of bee (*Apis mellifera*) venom Hyal (pdb_00001fcq) **(b)**. The Glyco_hydro_56 domains of Hyal from *Mesobuthus crucittii*, *O. martensii*, and *Apis mellifera* are showing in dark gray, cornflower blue, and purple, respectively. The EGF domain from *Mesobuthus crucittii* and *O. martensii* is showing in green. The cysteine residues in the Hyal of *Mesobuthus crucittii* and *O. martensii* are depicted in yellow, while the four homologous cysteine residues in *Apis mellifera* are highlighted in red. Summary of the domains from *Mesobuthus crucittii*, *O. martensii*, and *Apis mellifer* using a Simple Modular Architecture Research Tool (SMART) **(c)**.

To calculate the physicochemical properties and determination of the three-dimensional structure, we removed the 28-amino-acids signal peptide to keep only the 382 amino acids mature peptide. These analyses revealed that the Hyal of *M. crucittii* is a poor water-soluble protein with a molecular weight of 47500 g/mol. The Grand Average of Hydropathicity (GRAVY), instability index, aliphatic index, and Theoretical pI values measured −0.424, 37.58, 79.46, and 8.13 for Hyal, respectively. According to DbD2 web tool, the cysteine residues in the amino acids profile of Hyal have formed six disulfide bridges that stabilize the tertiary structure of this protein (Figure 4; Figure 5a; Supplementary Figure S2). To understand potential antibody-antigen interactions following the introduction of a protein antigen (such a scorpion Hyal), epitope identification is essential. We therefore predicted the antibody epitopes of *M. crucittii* Hyal using Bipipred Linear Epitope Prediction 2.0 (Supplementary Figure S5). This analysis identified 18 distinct epitope regions within the enzyme (visually represented in yellow, Supplementary Figure S5).

### 3.3 Molecular modeling

The three-dimensional structure of *M. crucittii* Hyal was modeled using AlphaFold, generating five initial models. Structural validation was carried out using the SAVES server tools (ERRAT, Verify3D, ProCheck), and the results of the quality assessment are summarized in Table 1. The model with the highest validation scores was selected for all subsequent analyses (Figure 5a); its quality was confirmed by a Ramachandran plot showing 94.3% and 99.7% of residues in the favored and allowed regions, respectively (Supplementary Figure S3).

**TABLE 1:** Structural evaluation results of the five 3D models generated by AlphaFold, based on SAVES server tools.

Model	ERRAT	Verify3D
1^*^	95.756	87.27
2	94.429	82.34
3	95.756	84.42
4	95.212	82.34
5	94.960	84.16

* the model with the highest validation scores, which was selected for all subsequent analyses.

Comparative structural alignment was performed between the selected *M. crucittii* model, its homolog from *O. martensii*, and the crystal structure from *Apis mellifera* (PDB: 1FCQ). This analysis revealed a high degree of structural conservation (Figure 5). Disulfide bond analysis indicated the presence of twelve cysteine residues forming six bonds in both scorpion structures, with an equal distribution between the Glyco_hydro_56 and EGF_3 domains. In contrast, the *A. mellifera* structure contained only four cysteine residues, which aligned homologously with a subset (C1, C3, C4, and C6) of the scorpion residues (Figures 5A,B; Supplementary Figure S4). Sequence alignment of Hyals from *M. crucittii*, *Olivierus martensii*, and *A. mellifera* is shown in Supplementary Figure S4. Domain analysis *via* InterProScan and SMART servers classified the *M. crucittii* enzyme within the Hyal and Glycosyl hydrolase 56 families, revealing two distinct domains: a catalytic Glyco_hydro_56 domain and an EGF_3 domain, which are shown in its structure (Figure 5C).

**FIGURE 6:**
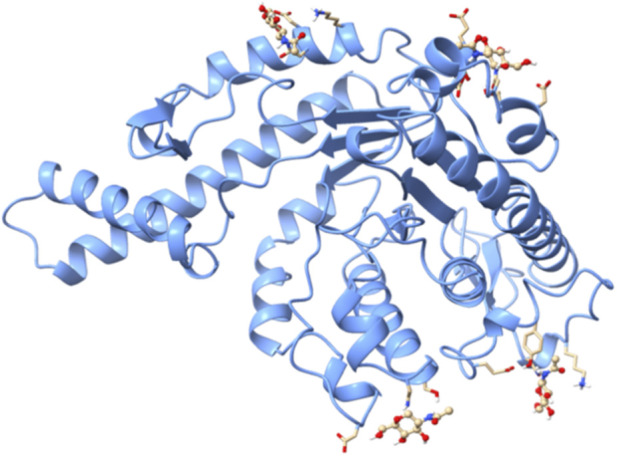
Predicted 3D structure of *Mesobuthus crucittii* Hyal. The structure was generated using the AlphaFold server (model rank 1). The four putative N-glycosylation sites, identified from the protein sequence, are highlighted in yellow.

Post-translational modifications (PTMs) for the *M. crucittii* Hyal sequence were predicted using the PTMGPT2 server (Shrestha e al., 2024). A comparative analysis of these predictions with UniProt annotations for the homologous protein (P86100) confirmed the conservation of four N-glycosylation sites at positions 48, 111, 256, and 392. To account for these modifications, a structural model was generated that incorporated all four conserved N-glycosylation sites (Figure 6). The amino acid region spanning residues 120 to 140 was selected as the putative ligand-binding site for docking simulations. This selection was based on the UniProt annotation for entry P86100, which identifies glutamate 130 (E130) as a catalytic residue, and on the presence of conserved catalytic motifs (GDWW, FPDC, and GWGS) identified in the current study. This region encompasses both the critical E130 residue and the GDWW catalytic motif, thereby increasing the likelihood of capturing key residues involved in substrate recognition and enzymatic activity.

### 3.4 Molecular docking

**FIGURE 7:**
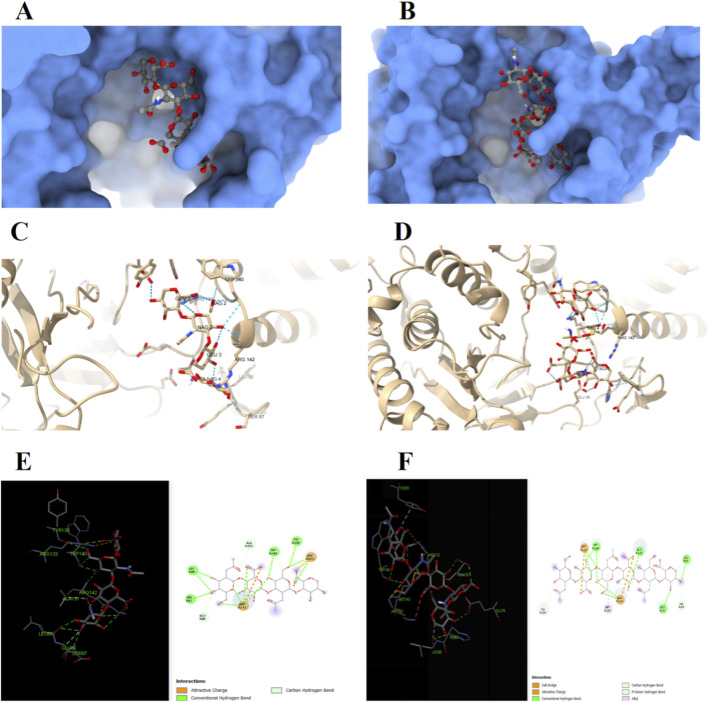
Molecular docking pocket, active site analysis and two-dimensional interaction diagram of hyaluronic acid tetrasaccharide **(A,C,E)** and hyaluronic acid hexasaccharide **(B,D,F)** with the Hyal enzyme.

**FIGURE 8:**
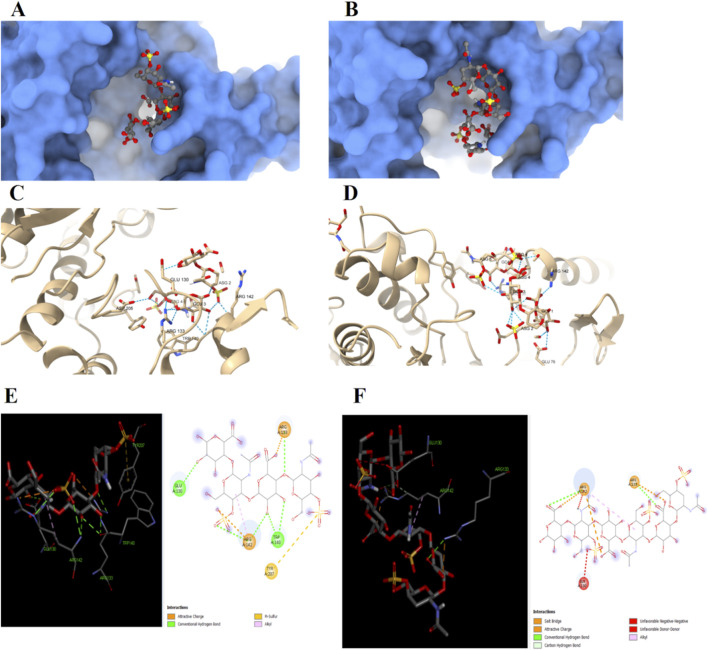
Molecular docking pocket, active site analysis and two-dimensional interaction diagram of chondroitin sulfate A tetrasaccharide **(A,C,E)** and chondroitin sulfate A hexasaccharide **(B,D,F)** with the Hyal enzyme.

To investigate the potential substrate binding affinity of Hyal, molecular docking was performed with two glycosaminoglycan substrates: hyaluronic acid (HA) and chondroitin sulfate A (CSA). The three-dimensional structures of the ligands were obtained from the Protein Data Bank (PDB). For hyaluronic acid, the hexasaccharide structure (PDB ID: 1HYA) was used, and a tetrasaccharide form was generated from this structure by removing the two terminal monosaccharide units. Similarly, for chondroitin sulfate A, the crystal structure (PDB ID: 1C4S) was used to generate a corresponding tetrasaccharide using the same method. The selection of tetrasaccharide and hexasaccharide forms for docking was based on the findings of Jiang et al. (2011), which identify these oligomers as the predominant hydrolysis products of Hyal activity. Consequently, a total of four ligands were docked into the enzyme’s catalytic site. The binding energy values and specific interactions observed in each docking simulation are presented in Table 2. The enzyme exhibited the highest binding affinity toward the tetrasaccharide form of hyaluronic acid, with a binding energy of −6.15 kcal/mol. This ligand also formed the highest number of hydrogen bonds with the enzyme. Key interactions observed across the complexes included hydrogen bonding, hydrophobic contacts, and electrostatic interactions. The molecular interactions between Hyal and each ligand are detailed in Figures 7, 8. R133 and R142 were consistently involved in hydrogen bonding and electrostatic interactions across all ligands, highlighting their critical role in substrate binding at the catalytic site (Table 2). Furthermore, certain ligands—with the exception of the tetrasaccharide form of hyaluronic acid—exhibited stronger interactions with the E130 residue at the catalytic site.

**TABLE 2:** Binding energies and interactions of Hyal with tetrasaccharide and hexasaccharide forms of hyaluronic acid and chondroitin sulfate A.

Ligand	Binding energy (kcal/mol)	Interaction type	Number of classical hydrogen bonds	Involved Amino Acids
HA tetrasaccharide	−6.15	Classical hydrogen bonding, C-H hydrogen bonding, electrostatic	11	L85, E86, S87, A131, R133, Y138, W140, R142
HA hexasaccharide	−4.58	Classical hydrogen bonding, C-H hydrogen bonding, electrostatic, salt bridge, van der Waals	9	E76, H84, L85, E130, R133, W140, R142, M143, Y209
CSA tetrasaccharide	−3.61	Classical hydrogen bonding, electrostatic, van der Waals, pi-sulfur	6	E130, R133, W140, R142, Y207
CSA hexasaccharide	0.38	Classical hydrogen bonding, C-H hydrogen bonding, electrostatic, salt bridge, van der Waals, repulsive interactions	2	E130, R133, R142

### 3.5 Hyaluronidase activity measurement

**FIGURE 9:**
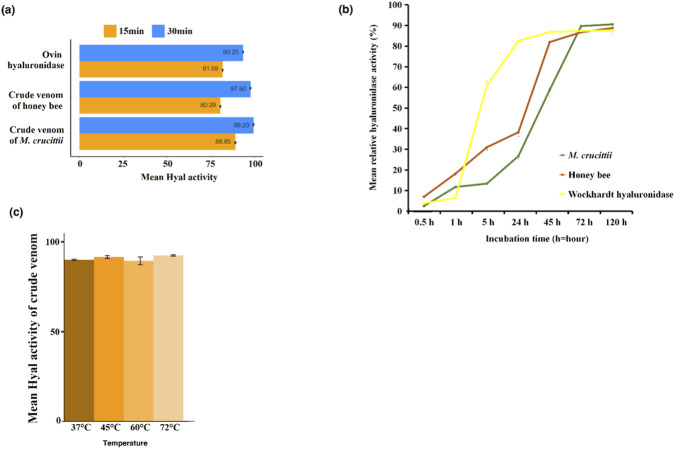
*Mesobuthus crucittii* Hyal activity. **(a)** Comparison of hyaluronan hydrolysis by *Mesobuthus crucittii* crude venom, bee venom, and ovine Hyal (as positive control); values represent the mean±SD of triplicate measurements (n = 3). **(b)** Hydrolytic activity against chondroitin sulfate of *Mesobuthus crucittii* crude venom, bee venom, and ovine Hyal. Data are presented as the mean±SD of triplicate assays (n = 3). Relative activity is shown only for *Mesobuthus crucittii*. **(c)** Thermal stability of *Mesobuthus crucittii* Hyal assessed after incubation at four different temperature, values represent the mean±SD of triplicate measurements (n = 3). Data are relative activity (%).

Quantitative analysis determined the protein concentration of *M. crucittii* crude venom to be 56.83 μg/mL. The hyaluronan-degrading activity of *M. crucittii* crude venom, honeybee crude venom, and a purified ovine Hyal standard was assessed by incubation with hyaluronan at 37 °C for 15 and 30 min (Figure 9a). It is important to note that the scorpion and honeybee enzymes were assayed as crude venoms without protein normalization, while the ovine enzyme was a purified standard. Therefore, this study was not designed to compare specific activities, and the ovine enzyme served solely as a positive control. The results demonstrated significant hyaluronan hydrolysis by *M. crucittii* venom at both time points. The same enzyme sources were also assayed for chondroitin sulfate-degrading activity over extended incubations (0.5–120 h) at 37 °C (Figure 9b). This experiment aimed solely to determine the capability of the scorpion and honeybee venoms to degrade chondroitin sulfate, not to quantify their activities. The results indicated that *M. crucittii* venom Hyal could degrade chondroitin sulfate, although this activity was substantially lower than its hydrolysis of hyaluronan.

The 120-h chondroitin sulfate incubation served two key purposes: (1) to establish the maximum stable incubation period for enzymatic assays by confirming the absence of spontaneous substrate degradation, and (2) to demonstrate sustained enzyme activity under prolonged experimental conditions. These results confirmed both the structural integrity of the substrate and the functional stability of the enzyme, thereby establishing optimal experimental parameters for subsequent investigations. Separately, to test the thermal stability of *M. crucittii* Hyal activity within whole venom, we assayed samples incubated at four different temperatures (37 °C, 45 °C, 60 °C, and 72 °C). We observed no significant change in mean Hyal activity after incubation at 60 °C (Figure 9C), indicating high thermal stability.

## 4 Discussion

### 4.1 Transcript diversity within the *Mesobuthus crucittii* venom gland

The study of scorpion venom composition has been facilitated by the use of transcriptomic analyses, with most of these studies focusing on highly toxic species of family Buthidae. Here, our high-quality venom gland transcriptomes of *M. crucittii* added to our knowledge on the composition of *Mesobuthus* venom. Although the venom of *Olivierus martensii* (=*Mesobuthus martensii*) has been extensively studied, that species has been transferred from *Mesobuthus* to *Olivierus* (Kovařík, 2019). Therefore, venom composition data currently exists for only three *Mesobuthus* species: *Mesobuthus eupeus* (Baradaran et al., 2018), *Mesobuthus gibbosus* (Diego-García et al., 2014), and *M. crucittii* (this contribution). Notably, our contribution represents the first to use RNAseq to analyze the venom composition within this genus. Our analysis reveals a diverse venom composition consistent with the diversity found in related scorpion species (e.g., Androctonus Salabi et al., 2021; Psenicnik et al., 2024; Zhang et al., 2015; Salabi and Jafari, 2024a; Salabi and Jafari, 2024b; Salabi et al., 2024; Possani et al., 1999; Santibáñez-López et al., 2022), *Centruroides* (Cid-Uribe et al., 2019; Samudio et al., 2025).

The venom of *M. crucittii* is characterized by ion channel toxins, enzymes, and other venom peptides. Specifically, the presence of transcripts with similarity to the lambda Potassium Channel Toxins (lKTxs) and chlorotoxins, cloned from the venom of *M. eupeus*, reinforces the hypothesis that these components are specific to buthid venoms (Santibáñez-López et al., 2018; Salabi et al., 2023a; Salabi et al., 2023b). To further explore this hypothesis, future studies should prioritize sampling additional species within the “Buthus” group and comparing their venom composition to those of other buthid lineages such as species in the “Tityus” and “Uroplectes” groups.

### 4.2 A new putative molecular diagnostic character for scorpions

Hyal or hyaluronoglucosaminidase, a member of the glycoside hydrolase family, is commonly found in animal venoms (Salabi and Jafari, 2023; Nazari et al., 2023; Nazari et al., 2024; Lüddecke et al., 2022; Erkoc et al., 2022; Pukrittayakamee et al., 1988). Scorpion venom Hyal, one of the acid-active enzymes, facilitates the spreading of venom components deeply into the bloodstream, which is critical in the systemic envenomation process (Marković-Housley et al., 2000). Some other functions including pore formation, cell membrane disruption, mast cell degranulation (dos Santos-Pinto et al., 2018), and increased permeability of blood vessels (Hossen et al., 2016) have also been related to this enzyme. However, this enzyme has little toxicity, and its role in venom is to facilitate the effects of other venom toxins (Bordon et al., 2015). Some isoforms of Hyal have been identified in the venom gland of some scorpion species, including *Tityus serrulatus* (Pessini et al., 2001), *Androctonus crassicauda* and *Hemiscorpius lepturus* (Salabi and Jafari, 2023), *Tityus bahiensis* and *Tityus stigmurus* (Guerra-Duarte et al., 2019), *Palamneus gravimanus* (Morey et al., 2006), *Buthus martensii* (Wali et al., 2023), and *Rhopalurus crassicauda* (Abreu et al., 2020). The transcriptome analysis of *Mesobuthus crucittii* venom glands exhibited low mRNA expression of Hyal that were in line with a previous study in the venom-gland transcriptome of *T. serrulatus* (Alvarenga et al., 2012), but in contrast with what Luna-Ramírez et al. (2015) reported. Our phylogenetic analysis suggests that Hyal genes have followed scorpion evolution, reflecting the split of the two parvorders (Buthida and Iurida), with the exception of one sequence from a buthid species nested within the Iurida clade. It is noteworthy to indicate that this sequence (P0C8X3 from *T. stigmurus*) is 24 amino acids long, which might suggest its spurious phylogenetic placement. Within the Buthida Hyal clade, we also observe an agreement between this gene tree and the scorpion tree of life (Santibáñez-López et al., 2022), with the major species groups recovered monophyletic (e.g., the “Buthus” group, “Tityus” group). Further analysis of these Hyal sequences would enhance the molecular characterization of these scorpion parvorders (Santibáñez-López et al., 2018).

### 4.3 Insights to scorpion venom hyaluronidase activity

Our *in silico* molecular characterization of the mature 382-amino acids Hyal form of *M. crucittii* predicted a poorly water-soluble 47.5 kDa protein with a hydrophilicity region (revealed by the negative GRAVY value), good structural stability (instability index lower than 40), and high thermostability (a high aliphatic index value) (Pondehnezhadan et al., 2023). Our computational prediction of *M. crucittii* Hyal (47.5 kDa) is consistent with *in silico* molecular weight estimates reported for other scorpion species, including *T. serrulatus* (∼45–48 kDa; Pessini et al., 2001), *Hottentotta tamulus* (∼47 kDa; Bordon et al., 2020), and *Androctonus australis* (∼46 kDa; Pondehnezhadan et al., 2023). However, experimental measurements *via* SDS-PAGE consistently demonstrate higher apparent molecular weights for these enzymes: 55–60 kDa (*T. serrulatus*), 65–70 kDa (*H. tamulus*), and 58–62 kDa (*A. australis*). This discrepancy between predicted and observed molecular weights (typically 50–75 kDa *versus* 45–50 kDa) primarily reflects post-translational modifications, particularly N-linked glycosylation, as confirmed by deglycosylation studies (Pessini et al., 2001; Abdel-Rahman et al., 2009). Furthermore, our motif analysis classified *M. crucittii* Hyal sequence as a member of glycosyl hydrolase family 56 (Baradaran et al., 2024; Salabi and Jafari, 2023; Baradaran and Salabi, 2023; Salabi et al., 2023b). Similar to what was reported for the Hyal from *T. serrulatus* (Bordon et al., 2015), we found that this enzyme contains a signal peptide (28 residues), catalytic sites, and twelve cysteine residues forming six disulfide bridges, which may reinforce the stability of the catalytic site (Clement et al., 2012). Here, we also reported three domains termed GDWW, FPDC, and GWGS as diagnostic conserved patterns in the sequences of scorpion venom-derived Hyals.

As reported before (Marković-Housley et al., 2000), scorpion venom Hyal facilitates the spreading of venom components during envenomation by degrading hyaluronic acid in the extracellular matrix of the skin matrix and the viscous polymer hyaluronic acid (hyaluronan) into non-viscous small hyaluronan oligosaccharides. This work focused on *in vitro* measuring the thermal stability and enzymatic activity of venom Hyal toward hyaluronic acid and chondroitin sulfate. Interestingly, we found that scorpion venom exhibits strong hyaluronan-degrading activity and, to a limited extent, chondroitin sulfate. Although chondroitin sulfate chains play a crucial role in various biological processes, the underlying mechanisms of their catabolism are not well known. Notably, *M. crucittii* the crude venom showed a lower apparent activity toward chondroitin sulfate compared to hyaluronic acid. This characteristic aligns with the activity profiles of bovine, ovine, and human Hyals (Honda et al., 2012; Stern and Jedrzejas, 2006). The observed activity toward both substrates is noteworthy, as the ability of snake venom Hyal to degrade chondroitin sulfate has been previously questioned (Kudo and Tu, 2001). Our findings, however, are consistent with earlier studies on the recombinant *Tityus serrulatus* Hyal, which also exhibited dual activity on hyaluronan and chondroitin sulfates (Amorim et al., 2018). Nevertheless, since our experiments were conducted using crude venom, further purification studies are required to confirm whether a single enzyme or multiple venom components are responsible for this dual activity in *M. crucittii*.

**FIGURE 10:**
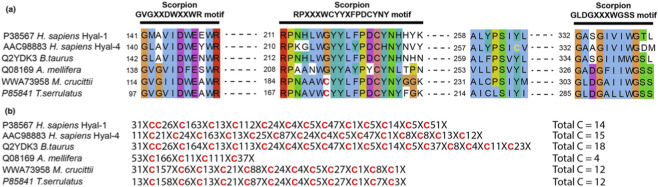
Molecular features of scorpion Hyals. **(a)** Multiple sequence alignment comparing the three scorpion domains suggested here in two human Hyals (Hyal-1 and Hyal4), a bovine Hyal, a bee Hyal, and two scorpion Hyal sequences (*Mesobuthus crucittii* and *Tityus serrulatus*). An additional comparative region is highlighted, pointing out the Cysteine residue in Human Hyal-4 (in yellow), and drawing attention to a Cysteine residue located four positions upstream in the scorpion sequences. **(b)** The number and position of each Cysteine residues in the Hyal sequences included in this comparison.

According to Stern and Jedrzejas (2006), the degradation of chondroitin sulfate by Human Hyal (Hyal-4, AAC98883) may stem from the presence of a Cysteine residue at position 264, which disrupts a conserved motif found in other Hyals, including bee Hyal. Here, we performed an *in silico* comparison of Hyal-4 against our scorpion Hyal sequences to identify key structural differences. While the scorpion Hyal retains the conserved Tyrosine (Y) in that position, we observed a Cysteine residue four positions upstream (Figure 10). The presence of this upstream Cysteine may represent a structural feature worth investigating in the context of substrate recognition (Honda et al., 2012; Stern and Jedrzejas, 2006). While a detailed *in-vitro* characterization of these differences is beyond the scope of this contribution, our *in silico* comparative analysis offers potential avenues for further research. For instance, the presence of disulfide bridges could significantly enhance protein stability. Bee Hyal sequence, for example, has the fewest Cysteine residues (only four) forming two disulfide bridges. In contrast, scorpion Hyal appears to be stabilized by six disulfide bridges, while mammalian Hyals are stabilized by seven and nine (Figure 10). Applied research with these molecules is often limited by the low quantities of native enzymes that can be isolated from the venom, making heterologous expression a promising alternative for potential biotechnological production (Amorim et al., 2018).

Lastly, our thermal stability assays of *M. crucittii* Hyal enzymatic activity suggested higher stability compared to other scorpion Hyals. For example, Ramanaiah et al. (1990) mention optimal activity for *Heterometrus fulvipes* Hyal at pH four and temperatures between 30 °C and 50 °C. Similarly, Morey et al. (2006) observed no loss of *Srilankametrus gravimanus* Hyal activity up to 37 °C, but a sharp decrease at 40 °C. In contrast, Pessini et al. (2001) found *T. serrulatus* Hyal activity to be optimal at pH 6 °C and 40 °C. However, Feng et al. (2008) reported an ideal temperature of 50 °C for *O. martensii* Hyal activity, which aligns closely with our observations. This similarity is particularly noteworthy given the close phylogenetic relationship between these two species (*O. martensii* and *M. crucittii*) compared to the others studied. Indeed, *O. martensii* and *M. crucittii* share 96% sequence identity, whereas the identity between *M. crucittii* and *T. serrulatus* Hyal is 72%. These sequence similarities may contribute to differences in protein stability or hydrophobicity, which may protect these enzymes against higher temperatures.

### 4.4 Oligosaccharide binding affinities

Structural studies on hyaluronate lyase have shown that tetrasaccharide substrates occupy fewer subsites within the active site cleft compared to hexasaccharide substrates, necessitating more extensive amino acid contacts to achieve stable binding and correct catalytic orientation. In contrast, longer hexasaccharide substrates distribute binding energy over additional sugar residues (occupying HA1, HA2, and HA3 subsites), thereby reducing the requirement for direct amino acid participation per sugar unit (Jedrzejas et al., 2002). This explains our observation that more amino acid residues participate in the catalytic site with tetrasaccharide than with hexasaccharide substrates. Consistent with this structural basis, the molecular docking results revealed that the venom Hyal from *M. crucittii* exhibits differential binding affinities toward hyaluronic acid and chondroitin sulfate A oligosaccharides. The HA tetrasaccharide demonstrated the most stable and favorable binding in the modeled glycosylated enzyme structure, characterized by the lowest binding energy and the most extensive hydrogen bond network. In contrast, the HA hexasaccharide, while forming stable contacts, displayed a lower binding affinity than the tetrasaccharide. Both CSA oligosaccharides showed weaker binding, with the hexasaccharide form exhibiting a positive binding energy and several unfavorable interactions, indicative of an unfavorable binding mode. The reduction in hydrogen bonds and the presence of these unfavorable contacts highlight the electrostatic and spatial incompatibility of CSA with the active site groove, likely due to its distinct geometry and high negative charge. Interestingly, the HA tetrasaccharide did not exhibit a direct interaction with the catalytic residue E130, despite its superior binding metrics. This suggests that binding stability is not solely dependent on direct contact with this residue. Instead, the initial binding phase may be driven by peripheral residues and an optimal spatial arrangement within the binding groove. Furthermore, transient interactions with E130, which are time-dependent, would not be captured by static docking and require dynamic investigation. Overall, the results suggest the enzyme has an optimal binding length, with the tetrasaccharide demonstrating the highest compatibility. Extending the chain to a hexasaccharide led to a relative decline in binding quality and a weakened hydrogen bond network. Longer, highly charged oligosaccharides are more susceptible to spatial and electrostatic incompatibility, while shorter chains likely fail to span the key interaction points within the active site. These findings align with previous reports (Jiang et al., 2011) identifying tetrasaccharides and hexasaccharides as the dominant hydrolysis products of Hyal, justifying the selection of these oligosaccharide lengths for comparison. They provide a coherent picture of the impact of ligand length and composition on binding efficiency. However, the inherent limitations of docking methods, such as the inability to capture solvation effects, protein flexibility, and temporal stability, necessitate complementary studies. Molecular dynamics simulations, free energy calculations, and experimental assays are required to confirm the exact role of catalytic residues, assess interaction stability, and explore the behavior of a broader range of oligosaccharides. Overall, despite their preliminary nature, these findings offer a coherent view of the enzyme’s binding preferences and establish a solid foundation for more detailed structural and dynamic investigations.

### 4.5 Limitations of the study

While the combination of transcriptomics, structural modeling, and functional assays provides a coherent first characterization of toxin complexity and hyaluronidase activity in *M. crucittii* venom, several limitations inherent to the current study design require careful consideration when interpreting the data.

A primary limitation concerns the attribution of enzymatic activity. All functional assays were conducted using crude venom rather than purified or recombinantly expressed hyaluronidase. Given the known complexity of scorpion venoms, which contain diverse enzymatic components including proteases, phospholipases, and other glycosidases (Delgado-Prudencio et al., 2022), the observed degradation of hyaluronic acid and chondroitin sulfate cannot be unequivocally assigned to the specific hyaluronidase transcript identified in the venom gland transcriptome. While hyaluronidases are well-established mediators of glycosaminoglycan degradation, the possibility that additional venom components contribute to or modulate the observed activity cannot be excluded. Consequently, the functional data presented here should be interpreted as evidence for hyaluronidase-associated activity within the venom rather than as a direct biochemical characterization of a single enzyme. Future studies employing protein purification or heterologous expression will be required to resolve the contribution of individual components and to establish specific activity profiles.

An additional limitation arises from the reliance on transcriptomic data for the identification of venom components. While venom gland transcriptomics represents a powerful approach to characterize toxin repertoires, it does not directly reflect the composition of the secreted venom (Smith and Undheim, 2018). Moreover, *de novo* venom gland transcriptome assemblies are prone to artefacts such as chimeric sequences, fragmented transcripts, and inflated diversity estimates, particularly in the absence of a reference genome (Walker et al., 2020). As a result, transcriptomic analyses may overestimate toxin diversity and may include transcripts that are not translated into functional venom components Walker et al., 2020). In the context of the present study, the identification of a putative hyaluronidase is therefore supported at the transcript level but remains to be validated at the proteomic and functional level. Integrative approaches combining transcriptomics with proteomics (“proteotranscriptomics”) and, where possible, genome-guided assemblies will be essential to confirm the presence, structure, and relative abundance of the identified enzyme within the venom.

A third limitation relates to the interpretation of molecular docking results. The docking analyses performed in this study provide a static representation of potential ligand-protein interactions and are therefore inherently limited in their ability to capture the dynamic nature of enzymatic catalysis. While the observed differences in binding energies and interaction patterns between hyaluronic acid and chondroitin sulfate oligomers suggest potential differences in substrate accommodation, these findings should be regarded as exploratory. More robust conclusions on substrate recognition and catalytic mechanisms will require complementary approaches such as molecular dynamics simulations, free energy calculations, or experimental structure–function analyses.

Finally, the designation of dual substrate specificity requires cautious interpretation. Although the crude venom exhibited detectable activity toward both hyaluronic acid and chondroitin sulfate, the experimental design was not intended to provide a quantitative kinetic comparison between substrates. The assays primarily demonstrate the capacity of the venom to degrade both glycosaminoglycans under the tested conditions, but do not allow rigorous assessment of substrate preference, catalytic efficiency, or turnover rates. As such, the term “dual activity” more accurately reflects the current data than “dual specificity,” which would require detailed enzymatic characterization including kinetic parameters (e.g., Km, Vmax) obtained from purified enzyme preparations.

## 5 Conclusion

Uncovering scorpion venom composition is important not only to understand venom evolution but to serve as a mine full of components with health applications. The study of venom composition through the use of RNAseq has transformed the discovery venom-gene candidates. However, while transcriptomes provide distinct advantages, these results should be validated using other data (genomics, proteomics, functional datasets implemented in AI pipelines). In the absence of high-quality genomes, conclusions on the number and presence/absence of toxins should be treated cautiously, as gene absence or low expression levels cannot be equated with gene loss or lack of expression. In this study, we combined transcriptomics and functional assays to successfully identify and digitally characterize a Hyal candidate from the venom of *M. crucittii*. Our work indicates that the crude venom is capable of degrading both hyaluronic acid and chondroitin sulfate, a feature reported for some but not all Hyals. Supporting these functional findings, our molecular docking analysis provides first structural evidence for the enzyme’s activity, suggesting a suitable binding affinity for hyaluronic acid tetrasaccharides. The framework established here paves the way for future dynamic simulations and experimental studies to fully realize this biotechnological potential.

## Data Availability

Raw data for venom gland transcriptomes were deposited into National Center for Biotechnology Information (NCBI) Sequence Read Archive (SRA) database under BioProject ID PRJNA1233588: Sequence Read Archive (SRA) accession SRR32918510, and SRR32918511, and Biosample accessions SAMN47273014, and SAMN47273015. The mRNA and protein sequences of *M. crucittii* Hyal reported in this paper are appearing in the NCBI under the accession numbers of PP347720 (mRNA sequence) and WWA73958 (protein sequence). Annotated transcripts are available upon request.
