# Supplementary material for: Venom gland transcriptomics and bioactivity profiling suggest bifunctional hyaluronidase activity in the venom of Mesobuthus crucittii (Scorpiones: Buthidae)
Source: Front Mol Biosci. 2026 Jun 1;13:1807239. doi: 10.3389/fmolb.2026.1807239 (PMC13294633; doi:10.3389/fmolb.2026.1807239)
Supplement: Supplementary file 1 [file Supplementaryfile1.docx]

**Supplementary Figures**

Baradaran et al. Venom Hyaluronidase from Iranian Endemic Scorpion *Mesobuthus crucittii* (Scorpiones: Buthidae): A breakthrough source for Next-Gen Drug Discovery


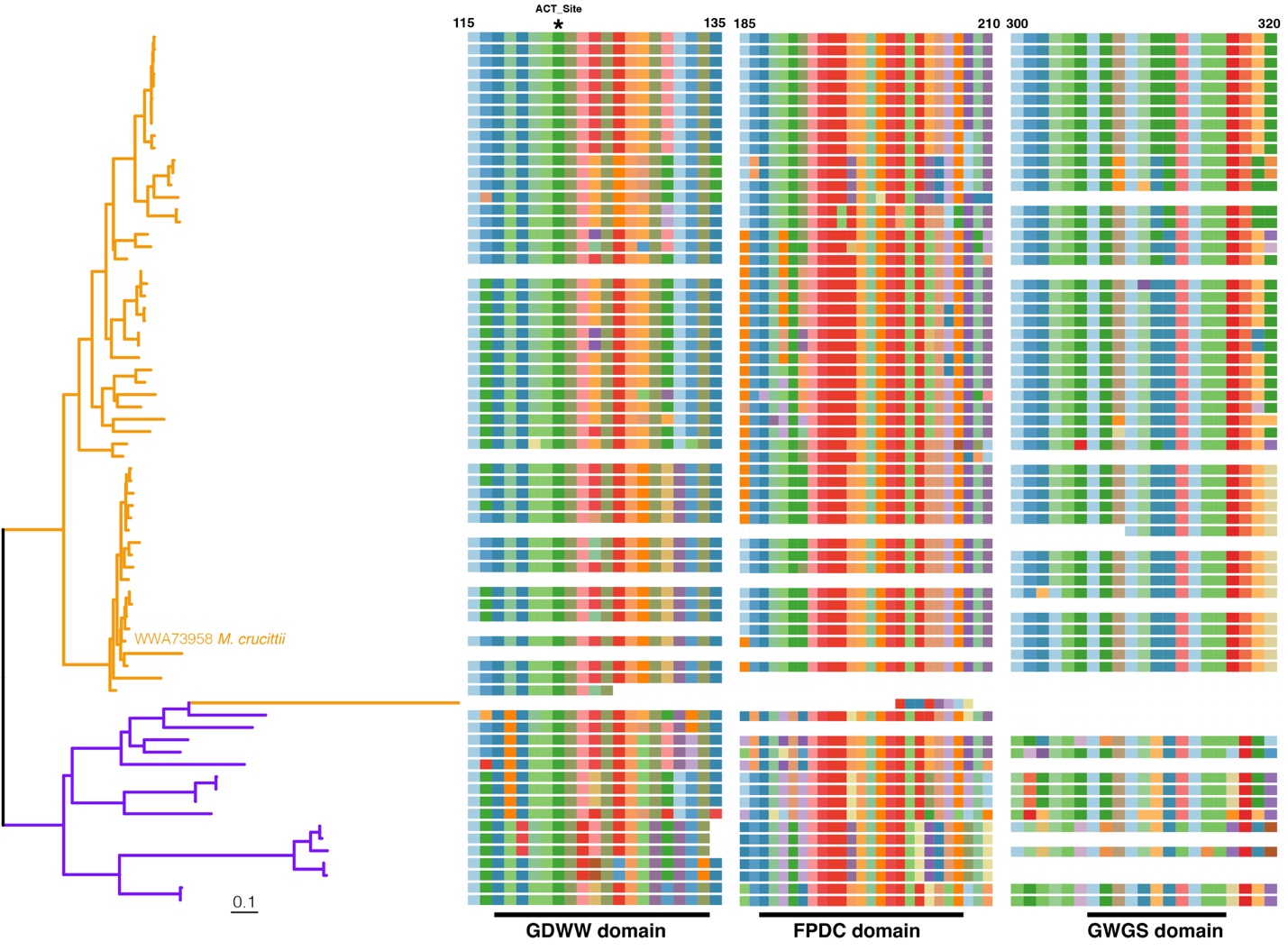


**Supplementary Figure S1.** Maximum likelihood gene tree of 71 scorpion hyualoronidase (Hyal) sequences. Orange branches indicate Buthida sequences, and purple branches indicate Iurida sequences. The multiple sequence alignment (MSA) showing the conserved GDWW, FPDC and GWGS domains is plotted alongside the phylogeny.


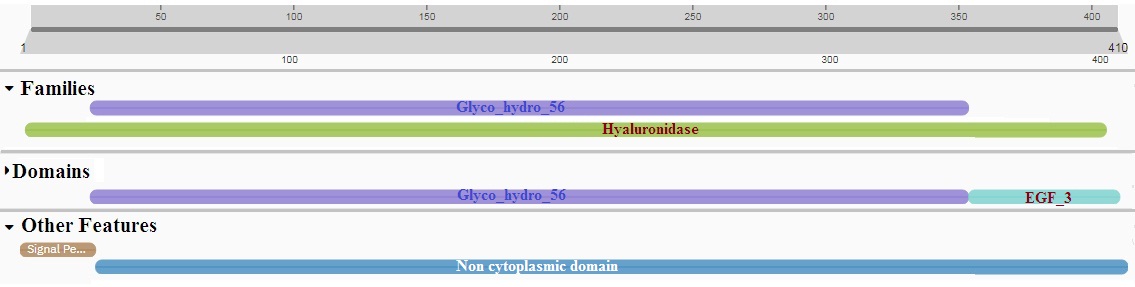


**Supplementary Figure S2.** Interproscan analysis of Hyal of *M. crucitti.*


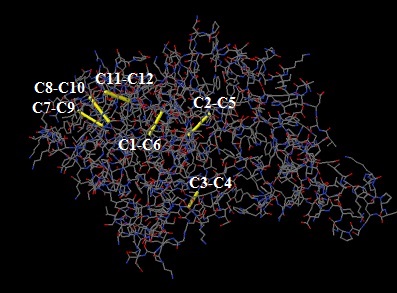


**Supplementary Figure S3.** Disulfide bonds of Hyal are represented in yellow. The predicted positions of Cysteines to form the disulfide bond are shown in the figure.


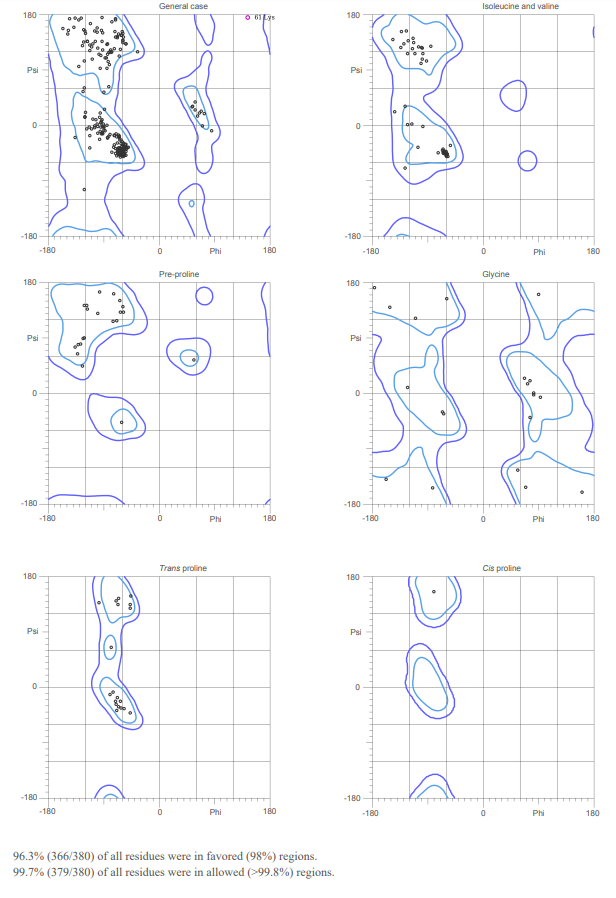


**Supplementary Figure S4.** Ramachandran plot for Hyal of *M. crucittii*.


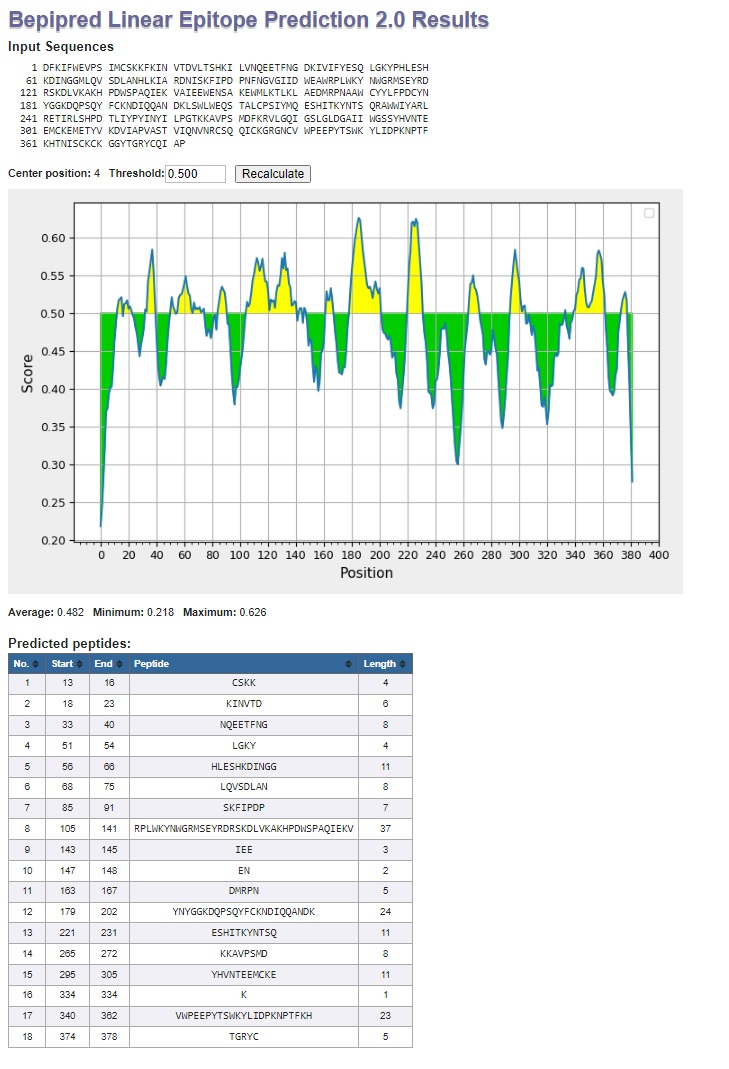


**Supplementary Figure S5.** Antibody epitope prediction [for](../../../../../../../../C:/Users/pc/Downloads/for) Hyal of *M. crucittii.* Epitope regions are shown in yellow and the sequence and exact position of them also illustrated in the table.
