# Supplementary material for: Venom gland transcriptomics and bioactivity profiling suggest bifunctional hyaluronidase activity in the venom of Mesobuthus crucittii (Scorpiones: Buthidae)
Source: Front Mol Biosci. 2026 Jun 1;13:1807239. doi: 10.3389/fmolb.2026.1807239 (PMC13294633; doi:10.3389/fmolb.2026.1807239)
Supplement: Supplementary file 2 [file Table1.docx]

**Supplementary Table**

Baradaran et al. Venom Hyaluronidase from Iranian Endemic Scorpion Mesobuthus crucittii (Scorpiones: Buthidae): A breakthrough source for Next-Gen Drug Discovery

**Supplementary Table S1.** List of the 71 scorpion Hase sequences used in our phylogenetic analysis.

| Parvorder | Family | Genus | Species | UniProt Accession No. or Reference | Tip label |
| --- | --- | --- | --- | --- | --- |
| Buthida | Buthidae | *Ananteris* | *balzani* | Santibáñez-López et al. (2022) | Abala_DN38413_c0_g1_i1p1 |
| Buthida | Buthidae | *Androctonus* | *bicolor* | A0A0K0LBS4 | A0A0K0LBS4_Androctonus_bicolor |
| Buthida | Buthidae | *Androctonus* | *crassicauda* | A0A7T9L322 | A0A7T9L322_Androctonus_crassicauda |
| Buthida | Buthidae | *Androctonus* | *amoreuxi* | Santibáñez-López et al. (2022) | Aamox_DN37671_c0_g1_i1p1 |
| Buthida | Buthidae | *Androctonus* | *bicolor* | Santibáñez-López et al. (2022) | Abico_comp9026_c0_seq1p1 |
| Buthida | Buthidae | *Androctonus* | *crassicauda* | Santibáñez-López et al. (2022) | AcrasII_trinity_DN183_c0_g1_i1p1 |
| Buthida | Buthidae | *Babycurus* | *gigas* | Santibáñez-López et al. (2022) | Bgiga_DN29606_c0_g1_i1p1 |
| Buthida | Buthidae | *Birulatus* | *israelensis* | Santibáñez-López et al. (2022) | Biisra_full_trinity_DN4143_c0_g2_i1p1 |
| Buthida | Buthidae | *Buthacus* | cf. *arenicola* | Santibáñez-López et al. (2022) | Baren_DN25787_c0_g1_i1p1 |
| Buthida | Buthidae | *Buthus* | *israelis* | Santibáñez-López et al. (2022) | Bisra_DN20629_c0_g1_i1p1 |
| Buthida | Buthidae | *Centruroides* | *hentzi* | A0A2I9LP21 | A0A2I9LP21_Centruroides_hentzi |
| Buthida | Buthidae | *Centruroides* | *caribensis* | Santibáñez-López et al. (2022) | Ccari_DN55027_c6_g1_i2p1 |
| Buthida | Buthidae | *Centruroides* | *sculpturatus* | Santibáñez-López et al. (2022) | Cscul_comp161284_c0_seq1p1 |
| Buthida | Buthidae | *Compsobuthus* | *leyvi* | Santibáñez-López et al. (2022) | Cleyv_DN33255_c0_g1_i1p1 |
| Buthida | Buthidae | *Compsobuthus* | sp. | Santibáñez-López et al. (2022) | CompsoI_trinity_DN9655_c0_g1_i1p1 |
| Buthida | Buthidae | *Compsobuthus* | *schmiedeknechti* | Santibáñez-López et al. (2022) | Cschi_DN30236_c0_g1_i1p1 |
| Buthida | Buthidae | *Grosphus* | *grandidieri* | Santibáñez-López et al. (2022) | Grosphus_DN18844_c0_g1_i1p1 |
| Buthida | Buthidae | *Heteroctenus* | *junceus* | Santibáñez-López et al. (2022) | Hjunc_DN46751_c0_g1_i1p1 |
| Buthida | Buthidae | *Heteroctenus* | *garridoi* | Santibáñez-López et al. (2022) | Rgarr_DN36922_c0_g1_i1p1 |
| Buthida | Buthidae | *Hottentotta* | *judaicus* | F1CIW6 | F1CIW6_Hottentotta_judaicus |
| Buthida | Buthidae | *Hottentotta* | *trilineatus* | Santibáñez-López et al. (2022) | Htril_comp160944_c0_seq1p1 |
| Buthida | Buthidae | *Isometroides* | *vescus* | T1DPA7 | T1DPA7_Isometroides_vescus |
| Buthida | Buthidae | *Jaguajir* | *agamemnon* | Santibáñez-López et al. (2022) | Jagam_DN10309_c0_g1_i1p1 |

**Supplementary Table S1** continue

| Parvorder | Family | Genus | Species | UniProt Accession No. or Reference | Tip label |
| --- | --- | --- | --- | --- | --- |
| Buthida | Buthidae | *Jaguajir* | *rochae* | Santibáñez-López et al. (2022) | Jroch_DN48262_c0_g1_i1p1 |
| Buthida | Buthidae | *Leiurus* | *hebraeus* | Santibáñez-López et al. (2022) | Lhebr_DN46609_c8_g1_i1p1 |
| Buthida | Buthidae | *Leiurus* | *quinquestriatus* | Santibáñez-López et al. (2022) | Lquin_DN28803_c0_g1_i1p1 |
| Buthida | Buthidae | *Lychas* | *variatus* | Santibáñez-López et al. (2022) | Lvari_DN45078_c0_g1_i1p1 |
| Buthida | Buthidae | *Mesobuthus* | *crucittii* | WWA73958 | WWA73958_Mesobuthus_crucittii |
| Buthida | Buthidae | *Olivierus* | *martensii* | P86100 | P86100_Olivierus_martensii |
| Buthida | Buthidae | *Olivierus* | *martensii* | Santibáñez-López et al. (2022) | Mmart_AYEL010873961 |
| Buthida | Buthidae | *Orthochiurus* | *scrobiculosus* | Santibáñez-López et al. (2022) | Oscro_DN31202_c0_g1_i1p1 |
| Buthida | Buthidae | *Parabuthus* | *transvaalicus* | Santibáñez-López et al. (2022) | Ptraa_comp56834_c0_seq1p1 |
| Buthida | Buthidae | *Tityus* | *bahiensis* | A0A0C9QKT6 | A0A0C9QKT6_Tityus_bahiensis |
| Buthida | Buthidae | *Tityus* | *bahiensis* | A0A0C9RFM5 | A0A0C9RFM5_Tityus_bahiensis |
| Buthida | Buthidae | *Tityus* | *obscurus* | A0A1E1WWG5 | A0A1E1WWG5_Tityus_obscurus |
| Buthida | Buthidae | *Tityus* | *serrulatus* | A0A218QWX6 | A0A218QWX6_Tityus_serrulatus |
| Buthida | Buthidae | *Tityus* | *serrulatus* | A0A218QX64 | A0A218QX64_Tityus_serrulatus |
| Buthida | Buthidae | *Tityus* | *serrulatus* | A0A218QX67 | A0A218QX67_Tityus_serrulatus |
| Buthida | Buthidae | *Tityus* | *serrulatus* | A0A7S8MU79 | A0A7S8MU79_Tityus_serrulatus |
| Buthida | Buthidae | *Tityus* | *serrulatus* | A0A7S8RGE3 | A0A7S8RGE3_Tityus_serrulatus |
| Buthida | Buthidae | *Tityus* | *melici* | A0AA49K9P2 | A0AA49K9P2_Tityus_melici |
| Buthida | Buthidae | *Tityus* | *melici* | A0AA49K9Q7 | A0AA49K9Q7_Tityus_melici |
| Buthida | Buthidae | *Tityus* | *melici* | A0AA49K9R1 | A0AA49K9R1_Tityus_melici |
| Buthida | Buthidae | *Tityus* | *melici* | A0AA49K9S4 | A0AA49K9S4_Tityus_melici |
| Buthida | Buthidae | *Tityus* | *melici* | A0AA49K9Y3 | A0AA49K9Y3_Tityus_melici |
| Buthida | Buthidae | *Tityus* | *melici* | A0AA49KA99 | A0AA49KA99_Tityus_melici |
| Buthida | Buthidae | *Tityus* | *stigmurus* | P0C8X3 | P0C8X3_Tityus_stigmurus |
| Buthida | Buthidae | *Tityus* | *serrulatus* | P85841 | P85841_Tityus_serrulatus |

**Supplementary Table S1** continue

| Parvorder | Family | Genus | Species | UniProt Accession No. or Reference | Tip label |
| --- | --- | --- | --- | --- | --- |
| Buthida | Buthidae | *Tityus* | *serrulatus* | W0HFN9 | W0HFN9_Tityus_serrulatus |
| Buthida | Buthidae | *Tityus* | *maranhensis* | Santibáñez-López et al. (2022) | Tarc_DN12308_c0_g1_i1p1 |
| Buthida | Buthidae | *Tityus* | *costatus* | Santibáñez-López et al. (2022) | Tcos_DN28770_c0_g1_i1p1 |
| Buthida | Buthidae | *Tityus* | *smithi* | Santibáñez-López et al. (2022) | Tsmi_DN2531_c0_g1_i1p1 |
| Buthida | Buthidae | *Troglorhopalurus* | *lacrau* | Santibáñez-López et al. (2022) | Tlac_DN24053_c0_g1_i1p1 |
| Buthida | Buthidae | *Uroplectes* | *olivaceus* | Santibáñez-López et al. (2022) | Uoli_DN40748_c0_g1_i1p1 |
| Buthida | Buthidae | *Uroplectes* | *vittatus* | Santibáñez-López et al. (2022) | Uvit_DN30975_c0_g1_i1p1 |
| Iurida | Bothriuridae | *Cercophonius* | *squama* | T1DEH9 | T1DEH9_Cercophonius_squama |
| Iurida | Euscorpiidae | *Megacormus* | *gertschi* | A0A224XBJ0 | A0A224XBJ0_Megacormus_gertschi |
| Iurida | Euscorpiidae | *Megacormus* | *gertschi* | A0A224XEV3 | A0A224XEV3_Megacormus_gertschi |
| Iurida | Euscorpiidae | *Megacormus* | *gertschi* | A0A224XFE2 | A0A224XFE2_Megacormus_gertschi |
| Iurida | Euscorpiidae | *Megacormus* | *gertschi* | A0A224XGH0 | A0A224XGH0_Megacormus_gertschi |
| Iurida | Euscorpiidae | *Megacormus* | *gertschi* | A0A224XGI5 | A0A224XGI5_Megacormus_gertschi |
| Iurida | Hadruridae | *Hadrurus* | *spadix* | A0A1W7RAD4 | A0A1W7RAD4_Hadrurus_spadix |
| Iurida | Hemiscorpiidae | *Hemiscorpius* | *lepturus* | A0A1L4BJ83 | A0A1L4BJ83_Hemiscorpius_lepturus |
| Iurida | Hemiscorpiidae | *Hemiscorpius* | *lepturus* | A0A7T9L375 | A0A7T9L375_Hemiscorpius_lepturus |
| Iurida | Superstitioniidae | *Superstitionia* | *donensis* | A0A1V1WBG3 | A0A1V1WBG3_Superstitionia_donensis |
| Iurida | Superstitioniidae | *Superstitionia* | *donensis* | A0A1V1WBG5 | A0A1V1WBG5_Superstitionia_donensis |
| Iurida | Superstitioniidae | *Superstitionia* | *donensis* | A0A1V1WBH0 | A0A1V1WBH0_Superstitionia_donensis |
| Iurida | Superstitioniidae | *Superstitionia* | *donensis* | A0A1V1WBI1 | A0A1V1WBI1_Superstitionia_donensis |
| Iurida | Superstitioniidae | *Superstitionia* | *donensis* | A0A1V1WBX2 | A0A1V1WBX2_Superstitionia_donensis |
| Iurida | Superstitioniidae | *Superstitionia* | *donensis* | A0A1V1WC22 | A0A1V1WC22_Superstitionia_donensis |
| Iurida | Urodacidae | *Urodacus* | *manicatus* | T1E6X0 | T1E6X0_Urodacus_manicatus |

**Supplementary Table S2.** Quality assessment of the *de novo* transcriptome assembly for *M. crucittii* venom gland using Trinity.

|  | | Stats based on all Transcript contigs | based on ONLY LONGEST ISOFORM per 'GENE' |
| --- | --- | --- | --- |
| Contig N10 | 5,591 | | 4,071 |
| Contig N20 | 4,115 | | 2,818 |
| Contig N30 | 3,219 | | 2,065 |
| Contig N40 | 2,524 | | 1,498 |
| Contig N50 | 1,968 | | 1,024 |
| Median contig length | 436 | | 335 |
| Average contig | 972.22 | | 634.80 |
| Total assembled bases | 200,425,098 | | 85,710,668 |

**Supplementary Table S3.** Quality assessment of the clustered *de novo* transcriptome assembly for *M. crucittii* venom gland using CD-HIT-EST.

|  | | Stats based on all transcript contigs | based on ONLY LONGEST ISOFORM per 'GENE' |
| --- | --- | --- | --- |
| Contig N10 | 4,400 | | 4,182 |
| Contig N20 | 3,058 | | 2,868 |
| Contig N30 | 2,199 | | 2,043 |
| Contig N40 | 1,582 | | 1,440 |
| Contig N50 | 1,061 | | 9,33 |
| Median contig length | 338 | | 331 |
| Average contig | 647.92 | | 614.56 |
| Total assembled bases | 63,121,168 | | 57,340,405 |

**Supplementary Table S4.** Summary statistics of *de novo* transcriptome assembly for *M. crucittii* venom gland.

|  | | Raw transcriptome | | Final transcriptome |
| --- | --- | --- | --- | --- |
| Total trinity genes | 135**,**021 | | 93,303 | |
| Total trinity transcripts | 206,153 | | 97,421 | |
| GC% | 32.81 | | 31.91 | |
